# Using operando techniques to understand and design high performance and stable alkaline membrane fuel cells

**DOI:** 10.1038/s41467-020-17370-7

**Published:** 2020-07-16

**Authors:** Xiong Peng, Devashish Kulkarni, Ying Huang, Travis J. Omasta, Benjamin Ng, Yiwei Zheng, Lianqin Wang, Jacob M. LaManna, Daniel S. Hussey, John R. Varcoe, Iryna V. Zenyuk, William E. Mustain

**Affiliations:** 1 Department of Chemical Engineering, University of South Carolina, Columbia, SC 29208 USA; 2 Department of Materials Science and Engineering; National Fuel Cell Research Center, University of California Irvine, Irvine, CA 92697-2700 USA; 3 Department of Chemistry, University of Surrey, Guildford, GU2 7XH UK; 4 National Institute for Standards and Technology, Gaithersburg, MD 20899 USA; 5 Department of Chemical and Biomolecular Engineering, University of California Irvine, Irvine, CA 92697-2700 USA

**Keywords:** Fuel cells, Fuel cells

## Abstract

There is a need to understand the water dynamics of alkaline membrane fuel cells under various operating conditions to create electrodes that enable high performance and stable, long-term operation. Here we show, via operando neutron imaging and operando micro X-ray computed tomography, visualizations of the spatial and temporal distribution of liquid water in operating cells. We provide direct evidence for liquid water accumulation at the anode, which causes severe ionomer swelling and performance loss, as well as cell dryout from undesirably low water content in the cathode. We observe that the operating conditions leading to the highest power density during polarization are not generally the conditions that allow for long-term stable operation. This observation leads to new catalyst layer designs and gas diffusion layers. This study reports alkaline membrane fuel cells that can be operated continuously for over 1000 h at 600 mA cm^−2^ with voltage decay rate of only 32-μV h^−1^ – the best-reported durability to date.

## Abstract (web summary)

Modern alkaline membrane fuel cells have generally shown very poor operational stability. Here, the authors combine operando neutron imaging and X-ray computed tomography to understand the root cause for this and then design new electrodes to enable high performance and operational stability.

## Introduction

Over the last decade, significant advances in the performance of alkaline membrane fuel cells (AMFCs) have been achieved in terms of peak power density and maximum current density^1,2^. More specifically, between 2018 and 2019 the development of thin alkaline exchange membranes (AEMs) with high ion exchange capacity (IEC) and high hydroxide conductivity^3–5^, and better understanding of water dynamics^6,7^ have enabled a dramatic boost in single cell AMFC performance.

On the membrane side, Varcoe et al. improved AMFC power density from 2.0 W cm^−2^ to 2.6 W cm^−2^ by switching from low-density to high-density polyethylene AEMs created by radiation-grafting^5^. Mandal et al.^4^ developed block copolymer ploy(norbornene) membranes that had ionic conductivity up to 200 mS cm^−2^ and could achieve unprecedented peak power density of 3.5 W cm^−2^ and maximum current density of around 10 A cm^−2^. Related to water management, Mustain et al.^6,7^ found that the AMFC peak power density and maximum current density can be extremely sensitive to operating conditions such as temperature as well as the dew points of both the anode and cathode, which was later verified by Wang et al.^8^. In addition, operando neutron imaging has shown that anode flooding is a common issue limiting AMFC performance and electrode optimization strategies can be used to mitigate anode flooding and improve performance^7^.

However, one challenge that has eluded AMFC researchers is long-duration cell operation (>1000 h) without significant degradation. As reviewed by Dekel, before 2018 there were very few reports of AMFCs that could be operated for more than 100 h at low current (~100 mA cm^−2^)^1^. Since then, the stability of AMFCs has improved, but still remains relatively low, with state-of-the-art cells typically being operated between 400–600 h at 600 mA cm^−2^ and either showing high degradation rates of around 600 μV h^−1^^7,9^, or experiencing dramatic voltage oscillations during the test due to poorly managed water^3,10^.

To help improve AMFC device stability, much of the literature to date has focused at the material level, which has led to the creation of AEMs with high alkaline stability (1000’s of hours in concentrated KOH with negligible degradation) and good mechanical properties^3,9,11–13^. The PtRu/C and Pt/C electrocatalysts that are deployed at the anode and cathode, respectively, are also known to be stable for over 1000’s of hours fuel cell operation^14^. Because the operational time is not enough to induce significant degradation of the catalysts or fully hydrated AEMs, the gap between the in cell performance longevity and ex situ component stability likely stems from poor control over operational variables and poorly balanced cell water. Excess anode water can lead to flooding and oscillations in cell behavior while insufficient water at the cathode can lead to ionomer degradation^15^. The properties of the anode and cathode electrodes can significantly affect the location and balance of cell water. Therefore, the catalyst layer composition, catalyst layer hydrophobicity, and gas diffusion layer (GDL) hydrophobicity can have a significant effect on cell performance and longevity. Unfortunately, these variables have not been systematically studied in operating AMFCs to date.

Here we show operando neutron imaging and operando X-ray computed tomography (CT) to study the AMFC water dynamics and catalyst layer structural transformation under various operating conditions. The results indicate that to achieve stable long-term AMFC operation, there must be a balance between electrode flooding and electrolyte degradation. To this end, we develop gas diffusion electrodes (GDEs) with hydrophobic components (PTFE) in both the GDL and catalyst layers. The end result is a high performing AMFC that also has record durability, which is shown through continuous operation for more than 1000 h at 600 mA cm^−2^ with a very low degradation rate.

## Results

### Effects of AMFC water on ionomer swelling and performance

**Fig. 1:**
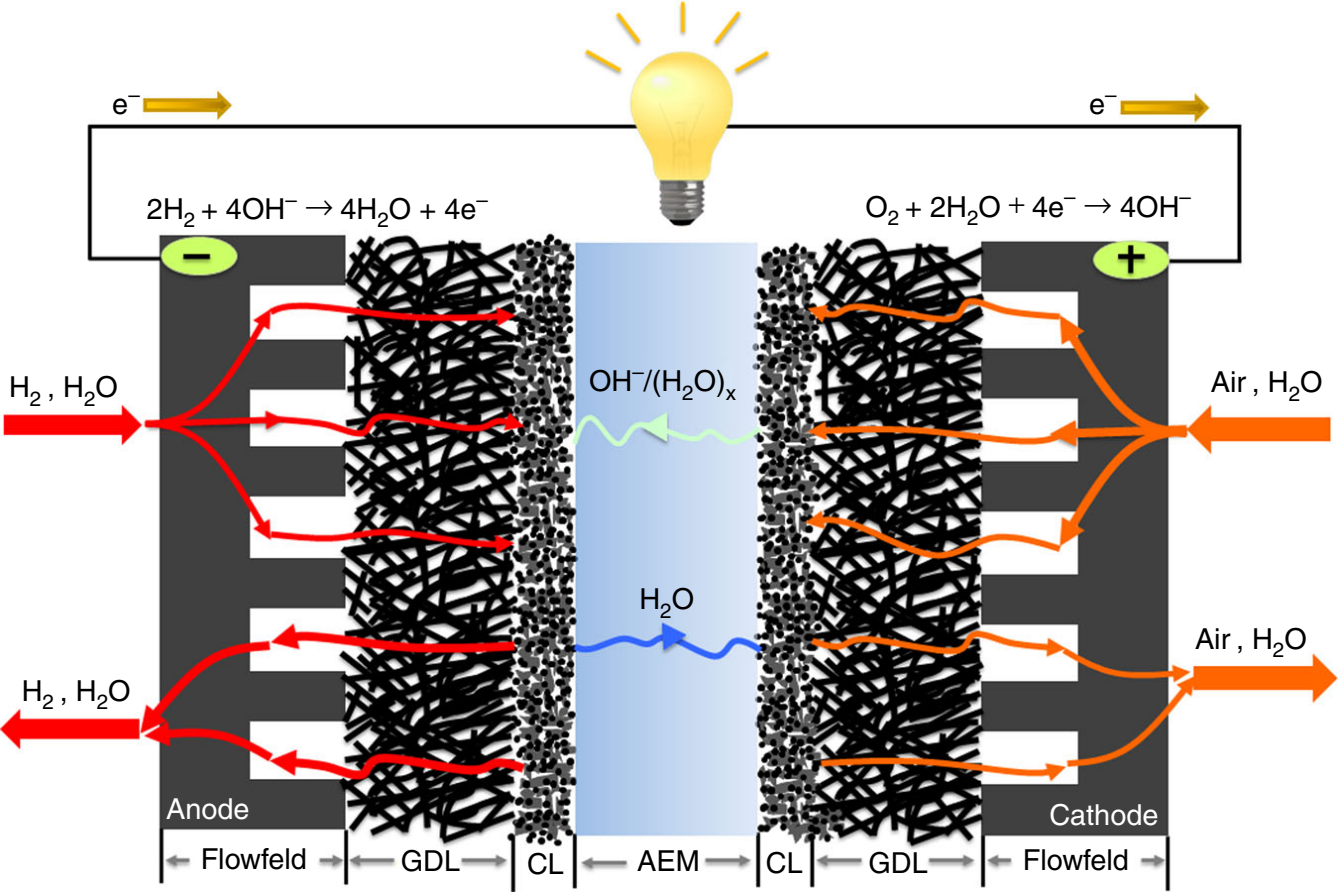
Illustration of AMFC water dynamics. The mass flows and electrode reactions define the water sources and sinks in an operating AMFC. In the figure, AEM = anion exchange membrane, CL = catalyst layer, and GDL = gas diffusion layer.

The water sources and sinks for an operating AMFC are shown in Fig. 1. Although the overall reaction is the production of two water molecules per reacting oxygen, water is both consumed (2 H_2_O/O_2_ at the cathode) and generated (4 H_2_O/O_2_ at the anode) in AMFCs. The product of the oxygen reduction reaction (ORR) at the cathode is OH^−^, which also completes the electrochemical circuit by moving from the cathode to the anode. As OH^−^moves through AEM, it has been estimated that at high levels of hydration up to eight water molecules move with each OH^−^ by electro-osmotic drag^16^. This is significantly more water transported than in the PEMFC, where no water is consumed, two H_2_O molecules are produced per reacting O_2_ at the cathode, and each proton only carries 1–2 water molecules toward the cathode by electro-osmotic drag^17^. Combined, the difference in reacting water and water flux by electro-osmotic drag makes the AMFC anode much more likely to flood than the PEMFC cathode, and it also makes the AMFC cathode much more likely to dry out than the PEMFC anode. Another complication is that the amount of H_2_O provided in the O_2_/air stream is not sufficient to support the ORR, especially at low flow rates and/or high currents. This means that the vast majority of the cathode reacting water in AMFCs must be supplied by back-diffusion through the AEM from the anode to the cathode^18^. To promote high water transport, high IEC polymers have been developed as AEMs and ionomers^19–21^.

**Fig. 2:**
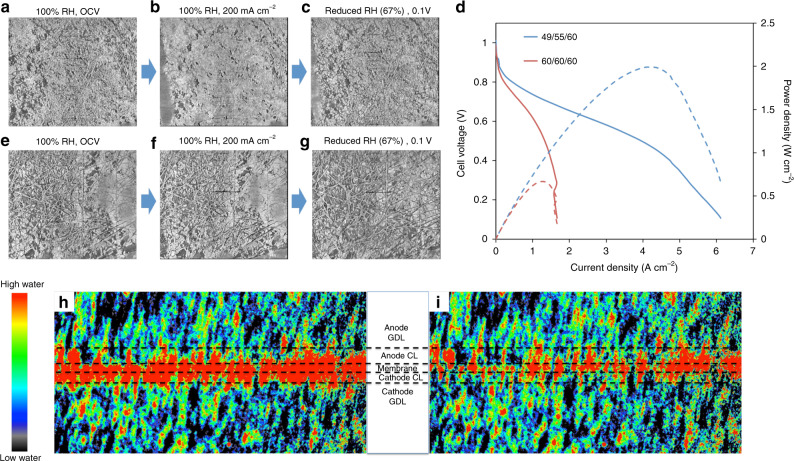
Dew point effects on electrode water and cell behavior. Operando micro X-ray computed tomography (CT) scans of the interface between the GDL and CL of (**a**–**c**) PtRu/C anodes and (**e**–**f**) Pt/C cathodes at conditions of: 100% RH at open circuit voltage (OCV), 100% RH at 200 mA cm^−2^, and reduced RH (67%) at 0.1 V, respectively. The H_2_/O_2_ flow rates were both kept at 0.2 L min^−1^. The cell temperature was kept at 28 °C (Polarization data for the operando cell is provided in Supplementary Fig. 5. **d** i-V (solid line) and i-power density curves (dashed line) at two different dew point configurations in 5 cm^2^ AMFCs. The membranes used in all tests were HDPE^5^. The 5 cm^2 ^ cells were tested at 60 °C with H_2_/O_2_ flows of 1 L/min. Operando neutron imaging of AMFCs at two different dew point situations: (**h**) The “full humidity” situation, which shows flooding inside the cell; (**i**) The “reduced dew points” situation, which shows drying out inside the cell. Anodes and cathodes used in the above three experiments had catalys loadings of 0.70 mg_PtRu_ cm^−2^ and 0.60 mg_Pt_ cm^−2^, respectively.

One of the downsides to increase the IEC is that the polymer typically also has higher water uptake, particularly in the presence of liquid water. Increased water uptake leads to ionomer swelling, which can have deleterious effects on cell behavior. More specifically, if the ionomer swells too much, there can be significant mass transport issues at the electrode level—both from flooding and reduced pore size. Figure 2a shows a cross-sectional X-ray CT image at the interface between the anode catalyst layer (CL) and gas diffusion lay (GDL) that has been equilibrated under gas flow at 100% relative humidity (RH). Additional ex situ and operando images of the catalyst layers are shown in Supplementary Figs. 1–4 and related discussion is provided in Supplementary Note 1-4. Though the ionomer can clearly be seen, a significant fraction of the cross-section is void space, which is needed to allow rapid gas/liquid transport. However, once current is allowed to flow and liquid water is produced, the ionomer in the catalyst layer swells significantly, Fig. 2b, nearly completely blocking the catalyst layer pores. This explains why in previous studies with the same ionomer^22^, the cell’s performance was very sensitive to the amount of water in the cell^6,7^. Interestingly, the ionomer has very high water uptake capability such that no liquid water is observed in the pores.

One of the methods that has been effective to enhance AMFC performance is to reduce the dew points of the reacting gases during operation^6,23^. This allows for convective evaporation of water into the anode flowfield, reducing the amount of water in the anode ionomer as well as the degree of ionomer swelling, Fig. 2c. As a result, the anode porosity is increased and the mass transport limitations on performance are alleviated, Fig. 2d, which also significantly increases the achievable peak power density^7^. This is true both in the 5 cm^2^ cells (Fig. 2d) as well as in the operando cells (Supplementary Fig. 5). Analysis of operando cell behavior during XCT experiments is provided in Supplementary Note 5. It should be noted that the achieved current and power density in the operando cells were lower than the 5 cm^2^ cells, due to differences in cell geometry that is needed to facilitate the imaging (and a very common tradeoff in X-ray CT and other operando techniques), but the trends were the same and the facilitated learning is directly applicable to other cell formats.

Under humidified gas flow at OCV (Fig. 2e), the cathode looks very similar to the anode (Fig. 2a), as expected. However, unlike the anode, when the cell is operated and current is drawn, the cathode does not undergo the same massive swelling as the anode, and under lower dew points (Fig. 2f) the cathode looks nearly the same as it did under humidified flow. However, it is possible to reduce the amount of water in the cathode to very low levels, either by reducing the oxygen feed dew point too low (Fig. 2g), significantly increasing the current density or having an AEM with insufficient ability for water back diffusion, which could sacrifice ionomer stability.

A complementary technique to study water dynamics in AMFCs is neutron imaging, which allows for the direct measurement and visualization of the amount of liquid water in operating cells. An AMFC was assembled with identical electrodes to the X-ray CT cell and imaged over various operating conditions. In this experiment, the cell was first operated at full humidity, Fig. 2h, where a significant amount of water was present in both the membrane and the electrodes. Next, the dew points of the reacting gases were simultaneously reduced, and the amount of liquid water in the cell decreased significantly, Fig. 2i, as the cell performance increased. A dynamic view of the reduction in cell water as the dew points are gradually reduced is shown in Supplementary Movie 1.

### Matching reacting gas dew points and AMFC operating current density

**Fig. 3:**
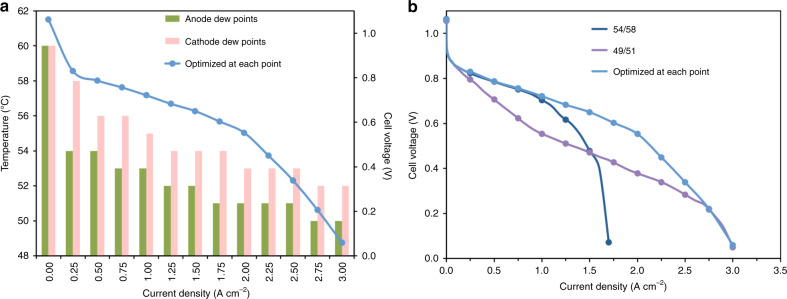
Current-dependent dew point optimization. **a** Polarization curve of a PtRu/C anode, Pt/C cathode AMFC with dew points optimized at each current density with a step size of 250 mA cm^−2^. Each condition was held for 30 min, (**b**) Comparison of polarization curves optimized at each point (30 mins/point) versus polarization curves where the dew points were set based on the optimum for a single current density, 3.0 A cm^−2^ (optimized dew points of 49 °C/51 °C) and 0.25 A cm^−2^ (optimized dew points of 54 °C/58 °C). The 49/51 curve was collected by stepping the current density backwards from 3 A cm^−2^ in 0.25 A cm^−2^ increments and the 54/58 curve was collected by stepping the current density forward from 0.25 A cm^−2^ in 0.25 A cm^−2^ increments. All conditions were held for 30 min.  The anode and cathode loadings were 0.70 mg_PtRu_ cm^−2^ and 0.60 mg_Pt_ cm^−2^, respectively. The cell operating temperature was 60 °C.

As described above, one of the keys to maximizing the performance of AMFCs is to balance the quantity of produced water at the anode with the amount that is removed by either convective evaporation or taken up by the membrane. Because lower current densities mean less water produced in the anode, less water consumed in the cathode and reduced water moving by electro-osmotic drag from the cathode to anode, it should be preferred to operate AMFCs at increasing reacting gas dew points as the current density decreases. This also means that operating at a single set of reacting gas dew points regardless of the current density is sub-optimal. To further examine the effect of the operating current density on the optimal gas feed dew points, a cell was assembled, exposed to the typical break-in procedure, equilibrated, and then the operating voltage was optimized while the cell ran at a constant current density of 3 A cm^−2^ by adjusting the reacting gas dew points. The resulting optimized dew points were 49 °C/51 °C (calculated RHs = 59%/65%) at the anode/cathode. The current density was then systematically decreased in 250 mA cm^−2^ steps, optimizing the dew points at each step. The equilibration time at each condition was no <30 min. These steps and the resulting optimized conditions are plotted in Fig. 3a, from which it can be seen that the optimal water balance is quite dependent on the current density, with the optimized gas feed dew points of 49 °C/51 °C at 3.0 A cm^−2^ to 54 °C/58 °C (calculated RHs = 75%/91%) at 250 mA cm^−2^.

To further demonstrate the current dependence, point-by-point polarization curves were taken at two sets of anode/cathode dew points: 54 °C/58 °C (the 250 mA cm^−2^ optimized dew point) and 49 °C/51 °C (the 3.0 A cm^−2^ optimized dew point). The resulting cell performances are compared with the AMFC polarization curve optimized at each point in Fig. 3b. It can be seen that the cases diverged significantly. At high stationary dew points (54 °C/58 °C), the cell was only capable of reaching 1.75 A cm^−2^ before experiencing premature flooding. With low stationary reacting gas dew points (49 °C/51 °C), membrane dry out, accompanied by an increase in the area specific resistance (ASR, Supplementary Fig. 6), limited performance. Also, holding the low dew points for an extended period of time at low water generation conditions was detrimental for the cell, causing irreversible performance loss due to cathode ionomer degradation.

**Fig. 4:**
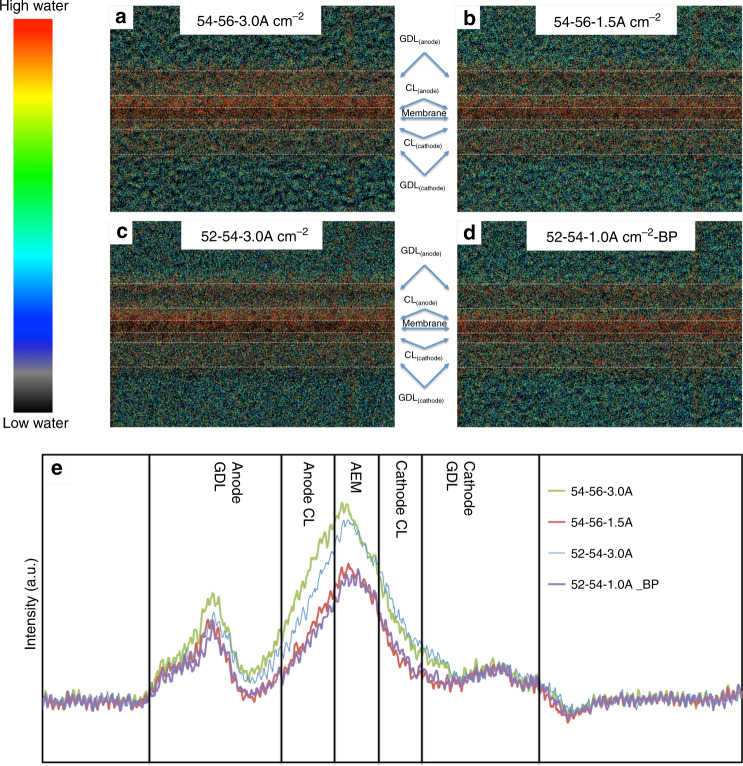
Water distribution at different operating conditions. Operando in-plane high-resolution neutron radiographic images of water in the gas diffusion layers, catalyst layers, and the radiation-grafted ETFE-BTMA AEM in operating at: (**a**) 3.0 A cm^−2^ with anode/cathode dew points of 54/56 °C; (**b**) 1.5 A cm^−2^ with anode/cathode dew points of 54/56 °C; (**c**) 3.0 A cm^−2^ with anode/cathode dew points of 52/54 °C; (**d**) 1.0 A cm^−2^ with anode/cathode dew points of 52/54 °C and 140 kPa back pressure; (**e**) qualitative through-plane water distribution plots extracted from high-resolution images. The intensity indicates the water thickness inside the MEA. The operando cells used in the neutron beam (1.2 cm^2^ active area) were constructed with a 0.70 mg_PtRu_ cm^−2^ PtRu/C anode, a Pt/C cathode at 0.60 mg_Pt_ cm^−2^, and a radiation-grafted ETFE-based AEI powder ionomer and ETFE-BTMA AEM (hydrated thickness = 50 μm). The cell temperatures were maintained at 60 °C. The full length of images are shown in Supplementary Fig. 7.

The relationship between the operating current density and dew points on the balance and distribution of cell water can be directly observed through neutron imaging. In Fig. 4, the operando AMFC was held at various conditions, each for more than 8 h. For two cells operating at different current density but the same reacting gas dew points (anode and cathode are 54 °C and 56 °C, respectively), a greater amount of liquid water is present in every part of the cell at higher current density (3.0 A cm^−2^, Fig. 4a) than lower current density (1.5 A cm^−2^, Fig. 4b). The difference in the total water content and distribution can also be observed from line scans, which are shown in Fig. 4e. It is counterintuitive that more water also accumulates at the cathode at higher current density, as a higher current density indicates more water is consumed via ORR and electro-osmatic drag at cathode. Therefore, this water accumulation at the cathode in Fig. 4a must come from water back diffusion through the membrane from the anode, driven by the water gradient due to high current density and therefore high water production rate.

At the same current density, 3.0 A cm^−2^, higher RH at both the anode and cathode led to more accumulated water in the membrane, CLs and GDLs at both anode and cathode. This can be observed by comparing the neutron images in Fig. 4a, c as well as the line scans in Fig. 4e. In fact, this comparison shows that the primary driver for cell water accumulation is the current density. Finally, back pressurization of the reacting gasses is a common technique that is used to enhance reactant activity, and hence reaction rate, in operating fuel cells. However, back pressurization can also influence the quantity and distribution of water in AMFCs by increases the total amount of vaporous water in the gas streams. Under relatively low anode/cathode dew points of 52 °C and 54 °C and low operating current of 1 A cm^−2^, the results of Fig. 3 would have suggested that the cell would experience dry out. However, applying symmetric back pressurization of 140 kPa (*P* = 240 kPa_abs_) to the anode and cathode, it was possible to achieve similar MEA-level water distribution (Fig. 4d) to higher RH, higher current density conditions (Fig. 4b) as shown in Fig. 4e. This makes sense because increasing back pressurization should reduce the rate of convective evaporation from the anode where liquid water is being produced. The dynamics of back pressurization are shown in Supplementary Movie 2, where the release of back-pressure (140 kPa → 0 kPa) led to a sudden rapid loss in MEA water paired with a change in the current density. Though not demonstrated here, it is also possible to increase cell performance through a combination of increased back pressurization and decreased reacting gas dew points.

### Impact of reducing dew points on AMFC durability

**Fig. 5:**
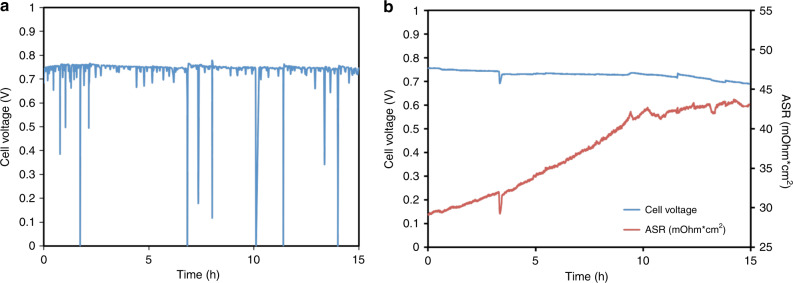
AMFC stability at high and low water conditions. **a** Cell stability under relatively high anode/cathode dew points of 55/60 °C; (**b**) cell stability under optimized anode/cathode dew points of 53/55 °C. During the test, the cell voltage and ASR were recorded. The cells were constructed with a PtRu/C anode at 0.70 mg_PtRu_ cm^−2^, a Pt/C cathode at 0.60 mg_Pt_ cm^−2^, and a radiation-grafted ETFE-based AEI powder ionomer and HDPE-based AEM^5^. The cell temperature was maintained at 60 °C. H_2_/air was kept at 1.0 L/min for anode/cathode, respectively. The cell current density was kept at 600 mA cm^−2^. The data is presented without iR-correction.

Another area where the water balance in the cell plays an important role is the operational stability. Figure 5a shows the behavior of an AMFC operating at 600 mA cm^−2^ at high anode/cathode dew points of 55 °C/60 °C—over a 15 h period following cell break-in. Though the cell voltage did not appear to decay significantly, even at this relatively modest current density the cell was very unstable due to frequent flooding events where the cell voltage would often drop nearly to 0 V. Of course, these events are untenable from an operation perspective and steps should be taken to eliminate them. As a result, one would naturally think to operate the AMFC at relatively low dew points to avoid catalyst layer flooding—as was done in Fig. 2g to improve performance. Therefore, the dew points of the reacting gases were iteratively optimized to achieve the maximum operating voltage at 600 mA cm^−2^ (anode/cathode dew points of 53 °C/55 °C). As shown in Fig. 5b, the cell did not experience any acute flooding events that forced the cell to low voltages. However, what did happen was that the observed voltage continued to drop over the duration of the experiment with an observed degradation rate of 2420 μV h^−1^. The most likely explanation for the cell degradation was irreversible polymer degradation due to the rapid increase in the ASR during the test (Fig. 5b). In AEM and AEI design, quaternary benzyl trimethylammonium is often used as the functional head group for hydroxide ion transport^3,5,11,22^. Density functional theory (DFT) simulations indicate that the energy barrier for nucleophilic attack by OH^−^ on the quaternary benzyl trimethylammonium is much lower under drier conditions than highly humidified conditions^15,24,25^, indicating the degradation rate of AEMs is much faster at low RH than that at high RH in operating AMFCs. However, the effect of low water and ionomer decay on the device behavior cannot simply be a reduction in conductivity because the ASR increase from 33.05 mOhm cm^2^ to 43.6 mOhm cm^2^ can only account for a 6 mV increase in the Ohmic overpotential at the tested current, while the AMFC experiences a voltage loss of ca. 90 mV during the test. It is also likely that polymer degradation also reduces the rate of water back diffusion through the AEM, lowering cell performance.

### Electrode modification to improve AMFC durability

The results in Figs. 2–4 suggest that there exists a balance between the amount of produced, transported, and removed water in operating AMFCs, and finding that balance results in the highest performance during polarization. However, Fig. 5 shows that the highest material durability exists at high water contents where operating cells are presently unstable and reducing the dew points of the reacting gases to balance water resulted in rapid polymer degradation. Therefore, there are two essential parts to be considered in terms of long-term stability tests: 1) suppressing polymer electrolyte degradation by operating at high reacting gas dew points while 2) preventing cell flooding. Considering these two factors, we propose a new GDE design for AMFCs that integrates hydrophobic components into both the catalyst layers and GDLs.

**Fig. 6:**
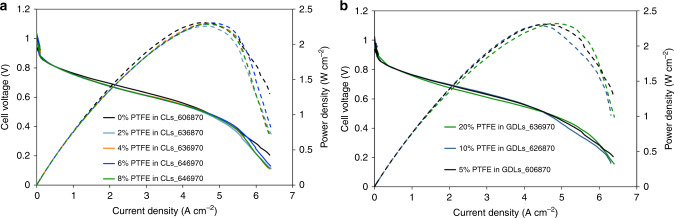
CL and GDL hydrophobicity effects on AMFC performance. **a** i-V and i-power density curves (10 mV s^−1^ forward scans) at optimal conditions in AMFCs. The CL PTFE was increased from 0 to 8 wt% with constant ionomer/carbon ratio of 0.4. The GDL PTFE content was kept constant at 5%; (**b**) i-V and i-power density curves (10 mV s^−1^ forward scans) at optimal condition in AMFCs. The GDL PTFE contents for both anode and cathode were tested at 5%, 10%, and 20%. The anode and cathode loading were 0.70 mg_PtRu_ cm^−2^ and 0.60 mg_Pt_ cm^−2^, respectively. The CLs PTFE mass content was kept at 0%. The membranes used in all tests were HDPE^5^. The cells were tested at 70 °C. The data is presented without iR-correction.

Anode and cathode catalyst layers with 0, 2, 4, 6, and 8 wt % PTFE were made. After break-in, the operating reacting gas dew points for each cell was optimized and the cell polarized. The results are shown in Fig. 6a. Increasing the PTFE content in the catalyst layers allows the cells to be operated at higher water content, with the optimized dew points continuously increasing as PTFE is added to the catalyst layer. Also one encouraging fact is that the polarization curves under optimized dew points were extremely similar and there was almost no impact on the peak power density. The only changes in the polarization curves are slight increases in the cell Ohmic resistance and lowering of the mass transport limiting current as the PTFE content is increased. The higher resistance at intermediate currents suggests that the electrodes reject more water to the anode flowfield and less to the AEM, which also likely is what leads to the lower mass transport limiting current since back-diffusion of liquid water across the AEM is rate-limiting, especially when high flowrate, pure O_2_ feeds are used. Next, the PTFE content of the anode and cathode GDLs were manipulated and catalyst layers were deposited on top of GDLs with 5, 10, and 20 wt% PTFE. Very similar trends in the polarization and power curves were observed when PTFE was added to the GDLs as when it was added to the catalyst layers directly, Fig. 6b. Therefore, to prevent cell flooding during operation at high reacting gas dew points, high PTFE content catalysts layers, and GDLs were used in AMFCs that were investigated both operando by X-ray CT and in long-term stability tests using conventional hardware.

**Fig. 7:**
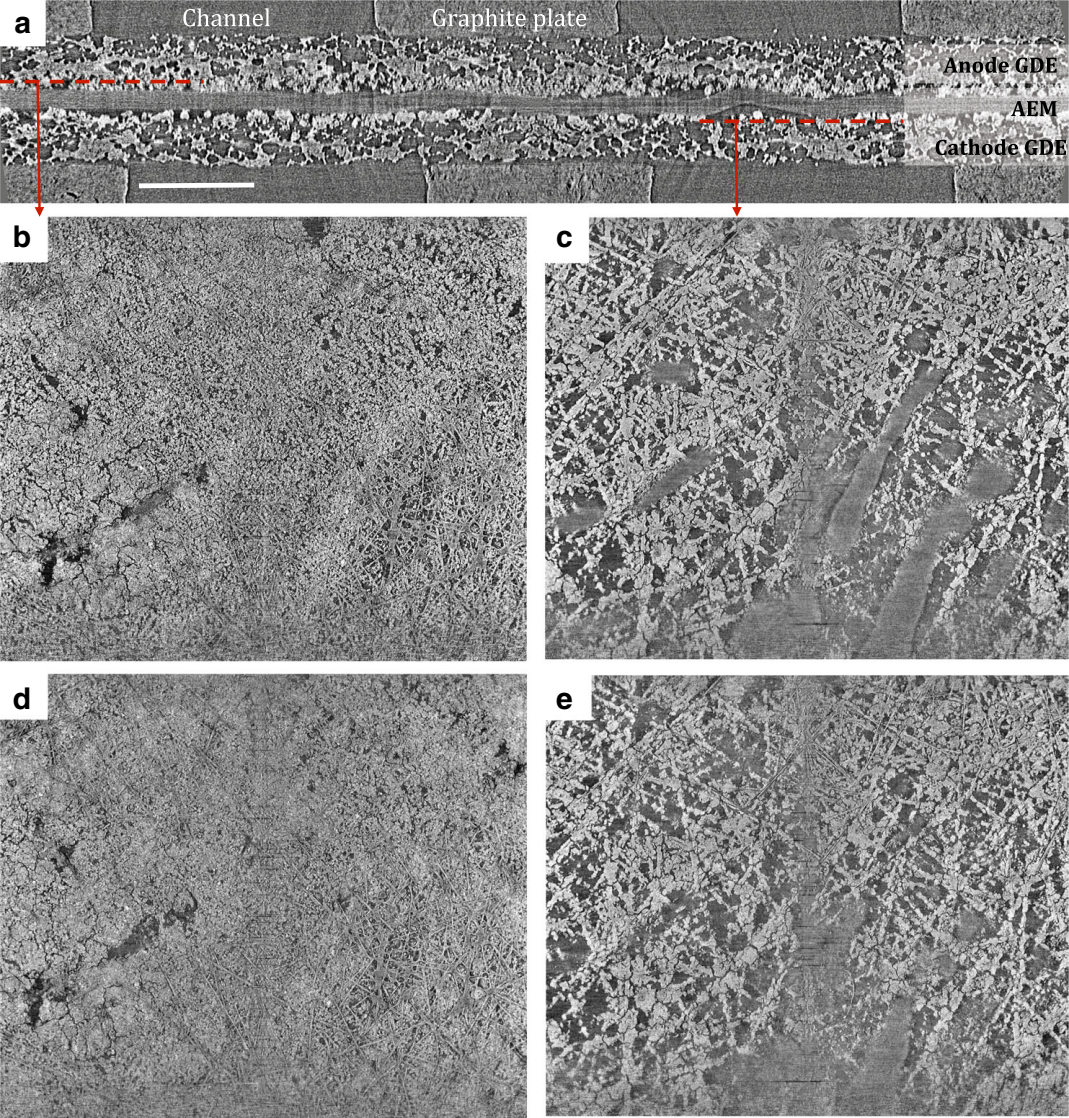
Morphology of electrodes with added PTFE. AMFC micro X-ray computed tomography images: (**a**) cross-section of the MEA showing the position of anode GDE, AEM, and cathode GDE of the imaged area; scale bar = 500 μm, (**b**), (**c**) longitudinal sections of anode and cathode GDEs at OCV under 100% RHs, (**d**), (**e**) longitudinal sections of anode and cathode GDEs at 0.1 V under 100% RHs. The anode and cathode used in this measurement contained 20% wt PTFE in the GDLs and 8% wt PTFE in the CLs. The anode and cathode loading were 0.70 mg_PtRu_ cm^−2^ and 0.60 mg_Pt_ cm^−2^, respectively. H_2_/O_2_ reacting gases were both fed to the cell at 200 sccm.

To verify the superiority of PTFE-added GDEs to manage water in operating AMFCs, operando X-ray CT images are shown in Fig. 7. The cross-section in Fig. 7a shows well-defined regions for the anode, AEM, and cathode, indicating the membrane electrode assembly (MEA) was well assembled in the tomography cell. Details regarding the experimental setup and data analysis are provided in the Methods Section^26–33^. At OCV under 100% RH, longitudinal sections of both the anode (Fig. 7b) and cathode (Fig. 7c) show very porous catalyst layers, indicating no visually observable flooding or ionomer swelling. Next, the cell was operated at 0.1 V in order to produce liquid water without reducing the dew points, similar to the conditions in Fig. 2. The current density vs. time plot is shown in Supplementary Fig. 8. However, unlike Fig. 2, during operation this electrode did not undergo significant physical changes and it appears that the ionomer swelling and water accumulation were successfully suppressed by integrating hydrophobic components in both CLs and GDLs, which is expected to be critical in achieving stable long-term operation.

**Fig. 8:**
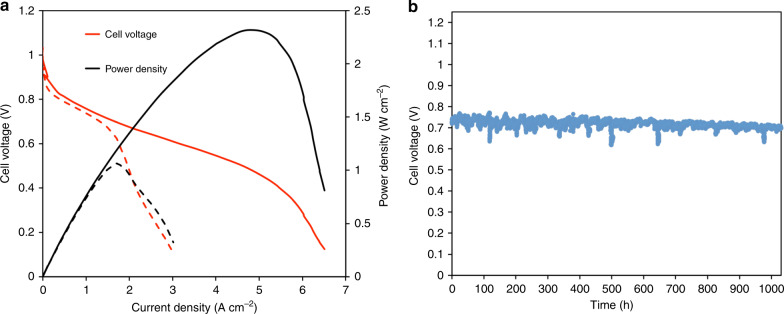
AMFC performance and stability using PTFE-added electrodes. **a** i-V and i-power density curves (10 mV s^−1^ forward scans, solid line H_2_/O_2_, dashed line H_2_/Air (CO_2_ free) at 1 L min^−1^) of an AMFC with 8% PTFE in the CL and 20% PTFE in the GDL for both the anode and cathode; (**b**) voltage vs. time at a constant current density of 600 mA cm^−2^ under H_2_/Air (CO_2_ free) flowed at 1 L min^−1^. The cell was held at 65 °C with both the anode and cathode RH set at 100%. The anode and cathode RHs were slightly adjusted to remove recoverable performance losses during the stability tests. The anode and cathode loading were 0.70 mg_PtRu_ cm^−2^ and 0.60 mg_Pt_ cm^−2^, respectively. The membrane used was HDPE^5^. The data is presented without iR-correction.

As shown in Fig. 8a, electrodes that are integrated with hydrophobic components were able to achieve peak power density of 2.35 W/cm^2^ under H_2_/O_2_ and 1.06 W/cm^2^ under H_2_/CO_2_-free air, corresponding very well to the state-of-the-art AMFC performance using similar cell components^5^. However, what is important here is that the electrode enabled extremely long-term stable operation. Under H_2_/air (CO_2_ free) gas flows at 65 °C, the AMFC was able to achieve stable, continuous operation for over 1000 h at 600 mA/cm^2^ with only 4.6% voltage loss (0.712 V → 0.679 V) during this time (Fig. 8b). The voltage degradation rate was only 32 μV h^−1^, a significant improvement over the state-of-the-art. The cell also did not experience any dramatic voltage oscillations. Throughout the test, the anode and cathode reacting gasses were maintained at around 100% RH, though they were occasionally slightly adjusted to remove recoverable performance loss due to fluctuation of the temperature in test stand humidifier bottles upon refilling. In addition to the voltage, the ASR was recorded throughout the experiment. The ASR (Supplementary Fig. 9) increased almost linearly at a rate of only 0.0157 mOhm cm^2^ h^−1^, indicating an excellent retention of the ion-conducting groups in the AEM. Compared with the stability data shown in Fig. 5, GDEs integrated with hydrophobic components were far superior, paving the way for new electrode designs and record AMFC durability. This data also shows that high stability AMFCs are possible, bringing them one step closer to overtaking PEMFCs in the low temperature fuel cell space.

## Discussion

This paper uses operando X-ray CT and operando neutron imaging in combination with electrochemical testing to understand the distribution of water in operating AMFCs under various operating conditions. It is shown that high RH operation is critical to achieve high stability; however, operating cells with high RH makes them susceptible to flooding. One way to assist the cell in balancing the rate of water produced by the cell and the rate of water rejection while maintaining high RH in the reacting gases is to redesign the electrodes, which is done here. Electrode designs that infiltrate the anode and cathode catalyst layers with hydrophobic PTFE are introduced for the first time in AMFCs and the amount of hydrophobic agent in the GDL is also studied. The results are AMFCs with operational stability that is significantly ahead of the current state-of-the-art AMFCs stability. This work shows the promising future of AMFCs for potential commercial applications and also provides knowledge that can be used to design non-PGM catalyst layers, design AMFC stacks and systems, and define operational variables for other research groups.

## Methods

### GDE fabrication and cell assembly

Prior to formulation of the catalyst ink, a ETFE-g-poly (VBTMAC) powder anionomer^22^ was first ground with a well-cleaned mortar and pestle for 5 min to reduce the number of aggregated particles. The anionomer has an IEC of 1.24 ± 0.06-meq g^−1^, water uptake of 155.4 ± 1.8%, and an average particle size of 24.5 ± 9.8 μm^22^. Next, the catalyst and 1 mL of DI water was added to the ground aniomer and ground for an additional 10 min until visually homogeneous catalyst slurry was formed. 40% Pt on Vulcan carbon (Alfa Aesar HiSPEC 4000, Pt nominally 40%wt, supported on Vulcan XC-72R carbon) and Pt-Ru on Vulcan carbon (Alfa Aesar HiSPEC 10000, Pt nominally 40%wt, and Ru, nominally 20%wt, supported on Vulcan XC-72R carbon) were used as electrocatalysts for cathode and anode, respectively. Extra Vulcan XC-72R carbon was added to the anode ink. The ETFE powder anionomer is comprised of 20% of the total solid mass of all of the GDEs in this paper. The ETFE ionomer to total carbon ratio was kept at 0.417 for both the anode and cathode. After the slurry was homogenized, 1.5 mL of isopropanol was added into the mortar followed by another 10 min grinding. A final 5 mL of isopropanol was added to the mortar and the final ink mixture was transferred to a PTFE- lined vial and sonicated for 1 h in ice bath. The prepared ink was then sprayed onto the GDL (Toary 60, Fuel Cell Store) using an air-assisted sprayer (Iwata) to fabricate GDEs. For the fabrication of PTFE (Ultraflon MP-25, Fuel Cell Store) treated electrodes, a measured amount of PTFE powder was directly added into the ground aniomer together with the catalyst and DI water and ground for 10 min until visually homogeneous catalyst slurry was formed before spraying.

### AMFC testing

AMFCs with 5 cm^2^ active area were assembled in single-cell hardware with a single channel serpentine flowfield. The anode GDE, cathode GDEs, and membrane were hydrated in DI water for 20 min and then soaked three times in 1.0 M KOH to remove impurities and ion exchange the quanternary ammonium groups before cell assembly. All membranes used in this study are listed in Supplementary Table 1. The average thickness of the anode and cathode were measured to be 203 μm and 205 μm, respectively. Thus, 152 μm Teflon gaskets were used on anode and cathode, respectively, to keep the pinch around 25% of the total GDE thickness. The AMFCs were tested on a Scribner 850e fuel cell test station at a cell temperature of 60 °C ± 0.1 °C under H_2_/O_2_ at 1.0 L/min. The temperature of the heated gas follow lines between the fuel cell test stand and the cell were maintained at 5 °C above the respective gas dew points. The cell was pre-operated at a voltage of 0.5 V for break-in, and the RH of both the cathode and anode was adjusted to help the cell to be operated at optimal conditions. Polarization curves were collected under potentiometric control at a scan rate of 10 mV/s. The cell was equilibrated for at least 5 min between taking different polarization curves after change of testing conditions.

### Neutron imaging cell and operation

The neutron scattering imaging experiments were conducted at the NIST Center for Neutron Research (NCNR)^34^. The experiment was built-in with PtRu/C anode and Pt/C cathode in an operando cell and analyzed using neutron imaging. The hardware has been previous described in detail^35^ and is summarized herein. Gold plated combination current collector and flow fields with a single serpentine flow pattern and active area of 1.2 cm^2^ were assembled with a Pt/C cathode and a PtRu/C anode. Both the ETFE-BTMA and PFAEM membranes were utilized with identical electrodes and assembled with 6-mil gaskets to achieve a 20% pinch. The cells were then humidified and broken in under the same protocol used for the 5 cm^2^ cells. It should be noted that the NIST cells are primarily optimized for the imaging techniques, and due to their smaller size, assembly requirements, and flow-field pattern (designed for optimal imaging of the electrodes and membrane), they do not achieve the same maximum performance as the Fuel Cell Technologies 5 cm^2^ hardware.

The neutron images and movies were collected on the BT- 2 beamline at the NCNR, and captured with a high-resolution CCD box with an MCP detector. For collimation, the L/D is 6000 along the through plane direction. Since the center of the test section was ca. 3 cm from the detector, the full-width half maximum of the geometric blur is ~2.5 μm. To align the fuel cell along the beam to yield 1 μm resolution would require an angular resolution on the rotation state of better than 0.005 degrees, which is within our state angular resolution of 0.001 degrees. For high-resolution measurements (data in Fig. 4), the spatial resolution of the detector is ≤1.5 μm^36^. For each of the conditions presented in Fig. 4, 400,000 images were recorded over several hours of operation and reconstructed through centroiding. Centroiding does significantly improved the spatial resolution—for conventional images without centroiding the scintillator resolution is about 20 μm, which is approximately the spatial resolution^37^—but the tradeoff is that recorded images lack the blending observed with lower resolution. Hence, the images in Fig. 4 look more pixilated than lower resolution images despite having much higher spatial resolution. The solution to resolve pixilation is to take more frames; we estimated that achieving a completed water map for each condition would have taken our team more than 1 million frames, which our teamtime allocation did not allow. Despite this, the results in Fig. 4 remain representative of the true water distribution in the cell and quantitative. For each data point of conventional neutron images, 20 images were averaged, combined, deconvoluted, and analyzed using NCNR software & protocols. The conditions that were varied during these experiments were the feed stream dew points and the current density.

### X-ray CT imaging

AMFC tomography images were acquired at the Advanced Light Source (ALS), Lawrence Berkeley National Laboratory. Image acquisition was conducted at x-ray CT Beamline 8.3.2 using a 50 μm LuAg:Ce scintillator, 5× lenses, and a sCMOS PCO Edge camera. Images resulted in a voxel resolution of 1.3 μm and a horizontal field of view of 3.3 mm. A double mulitlayer monochromator was used to select a beam energy of 24 keV. With 200 ms per projection the total scan time was 4 min.

The operando hardware used for micro x-ray CT imaging was reported previously^27,28^. Briefly, the design is summarized here. Due to high x-ray transmission and electric conductivity, graphite was chosen for the bipolar plates. The bipolar plates had two parallel flow channels with 1 mm × 1 mm dimension; the channels were separated by 1 mm. The active area of the MEA was 1 cm^2^, however, the imaged area was 3.3 × 3 mm. Kapton film was used to mask the area of 1 cm^2^. Aluminum clamps and bolts were used to compress the MEA and teflonized fiber-glass hard-stop gaskets were used to compress the GDEs to 20%. Two cartridge heaters were used for cell heating, with temperature control regulated by a proportional-integral differential controller.

For the micro CT operando experiments H_2_ and O_2_ were fed on anode and cathode at 0.2 slpm. The imaging was conducted either on constant current density (to produce the same amount of water) or if the cell showed instability due to flooding or drying out and couldn’t maintain the potential above zero for constant current operation then constant potential was selected. Imaging was also conducted at OCV condition as a reference image. Before imaging several polarization curves were collected. An EIS test and polarization curves were taken after every tomography scan. A frequency range from 500 kHz to 100 mHz was used with a 5 mV AC perturbation for the EIS experiments.

To create the X-ray CT images, a three-dimensional image stack is generated from a combination of two-dimensional x-ray projections. The reconstructions and phase retrieval were performed using the Gridrec algorithm^29,30^ with open-source TomoPy^31^. The reconstruction parameters and details are described previously^32,38^. Image processing, 8-bit conversion, and analysis was carried out with open-source Fiji/ImageJ^33^.

## Supplementary information

Supplementary Information

Peer Review

Description of Additional Supplementary Files

Supplementary Movie 1

Supplementary Movie 2

## Data Availability

The data that supports the plots within this paper and other findings of this study are available from the corresponding author upon reasonable request.

## Code availability

The code that supports the data processing within this paper and other findings of this study are available from the corresponding author upon reasonable request.
