# Supplementary material for: Using operando techniques to understand and design high performance and stable alkaline membrane fuel cells
Source: Nat Commun. 2020 Jul 16;11:3561. doi: 10.1038/s41467-020-17370-7 (PMC7366663; doi:10.1038/s41467-020-17370-7)
Supplement: Supplementary file 1 — Supplementary Information [file 41467_2020_17370_MOESM1_ESM.pdf]

Supplementary Information:

Using *operando* techniques to understand and design high performance and stable alkaline membrane fuel cells

Peng et al.

Supplementary Figures

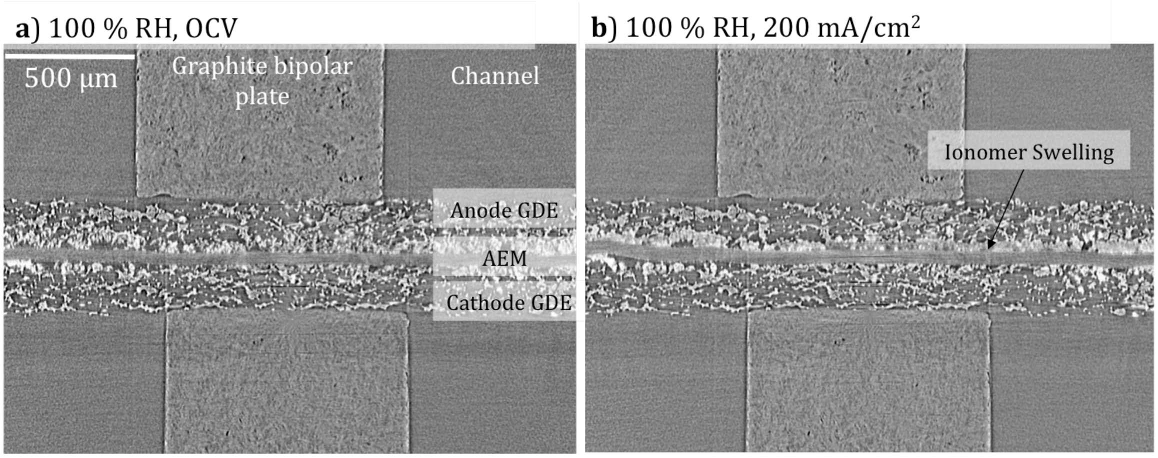

Supplementary Figure 1. Cross-section X-ray tomograph of AMFC with micro X-ray CT. Anode, cathode GDEs, membrane, bipolar plate and channels are marked. a) Cross-section at 100 % RH OCV condition, b) 67% RH at 0.1 V, respectively. H_2/O_2 flow rates were kept at 0.2/0.2 L min^{-1}, respectively.

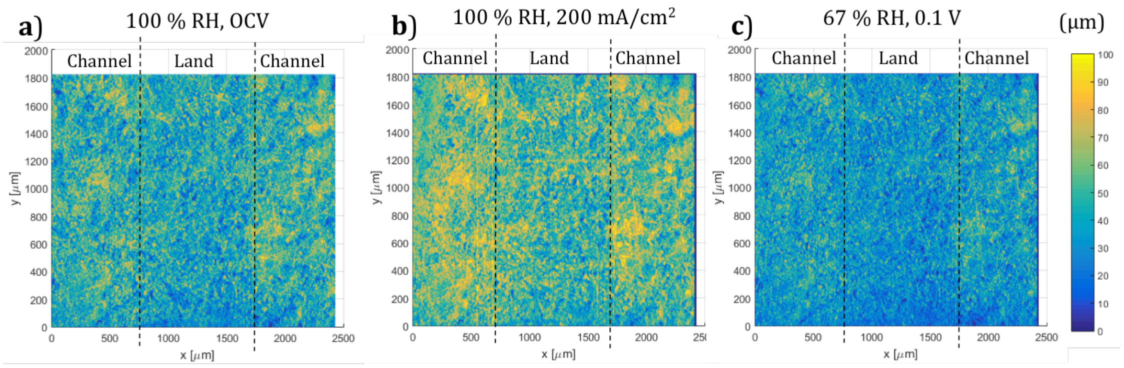

Supplementary Figure 2. Operando through-plane combined water and carbon thickness for a) 100 % RH, OCV, b) 100 % RH and 200 mA/cm^2 and c) 67 % RH and 0.1 V operating conditions. The land and channel domains are marked too.

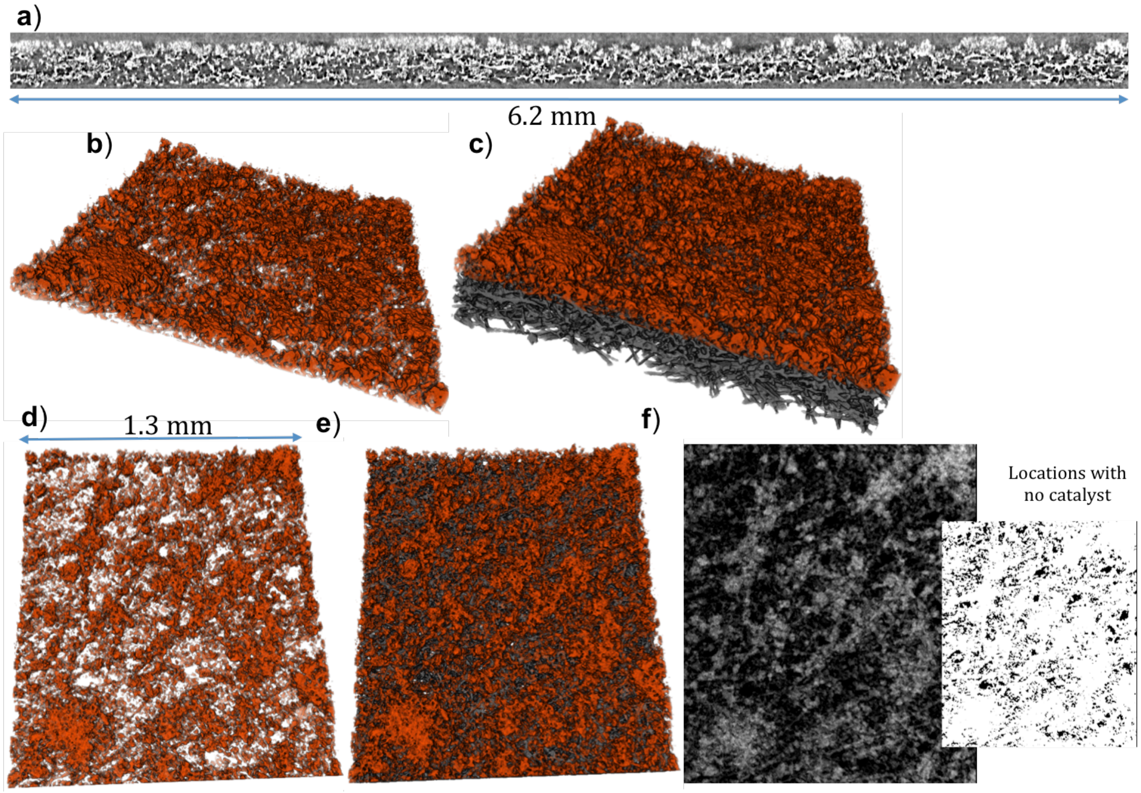

Supplementary Figure 3. Micro X-ray CT grey-scale tomograph and volume rendering of PtRu/C anode GDE. a) Cross section; b) catalyst distribution visualization without showing gas diffusion layer; c) catalyst distribution visualization showing gas diffusion layer; d) top view of b); e) top view of c); f) location with no catalyst, indicated from the dark area.

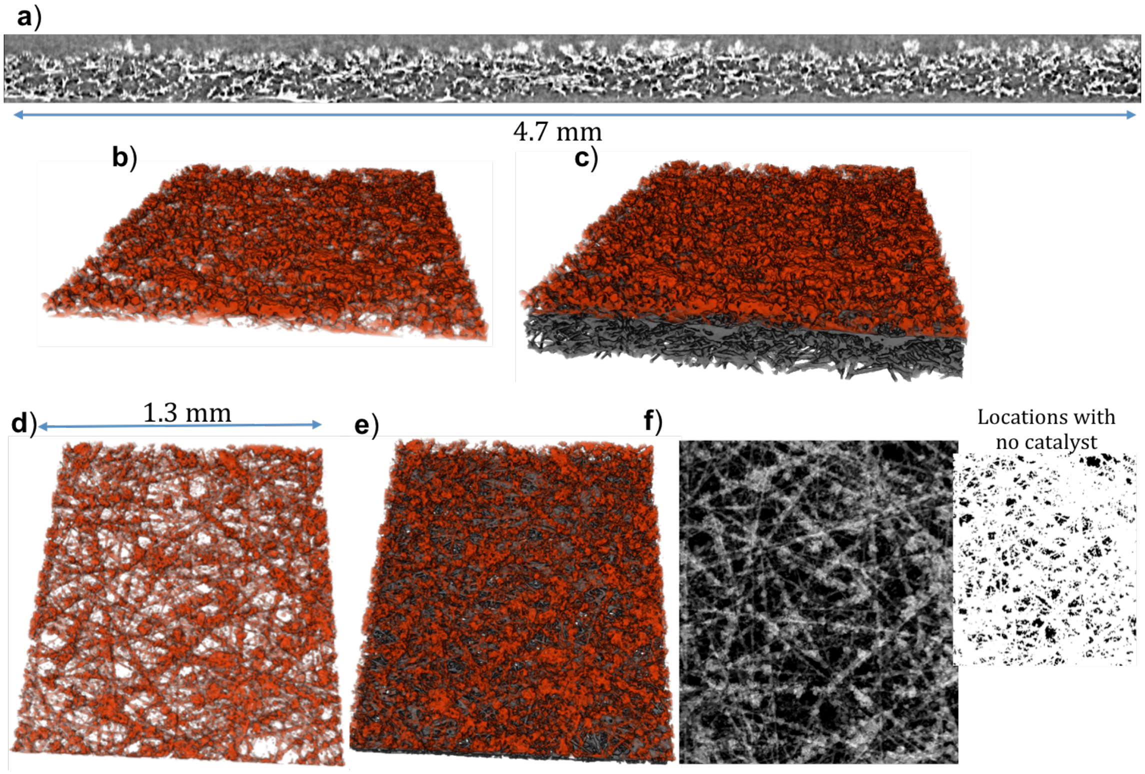

Supplementary Figure 4. Micro X-ray CT grey-scale tomograph and volume rendering of the Pt/C cathode. a) Cross section; b) catalyst distribution visualization without showing gas diffusion layer; c) catalyst distribution visualization showing gas diffusion layer; d) top view of b); e) top view of c); f) location with no catalyst, indicated from the dark area.

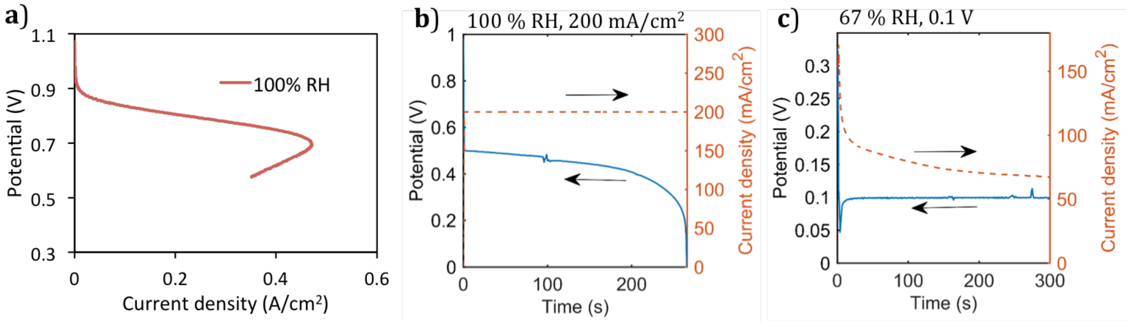

Supplementary Figure 5. a) Polarization curves (not iR-corrected) from operando X-ray CT for 100 % and 67 % RH, cell 28°C, H_2/O_2 flow rates were kept at 0.2/0.2 L min^{-1}. b) Constant current density operation at 100 % RH, c) constant potential of 0.1 V operation for 67 % RH. Plot a was collected after X-ray CT scan, plots b and c are collected during X-ray scan, which was about 4 minutes long.

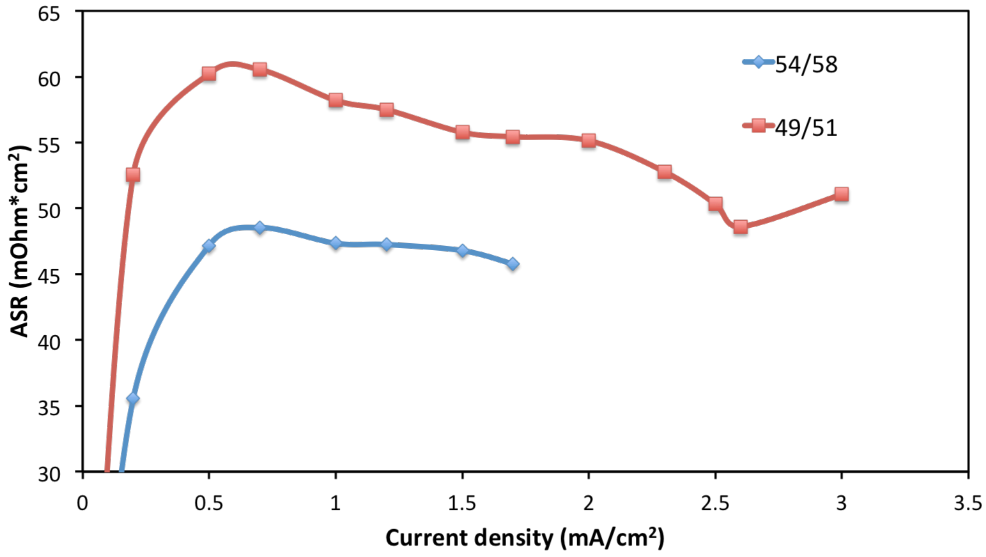

Supplementary Figure 6. Area specific resistance (ASR) that was recorded during the point-by-point polarization curves in Figure 3b.

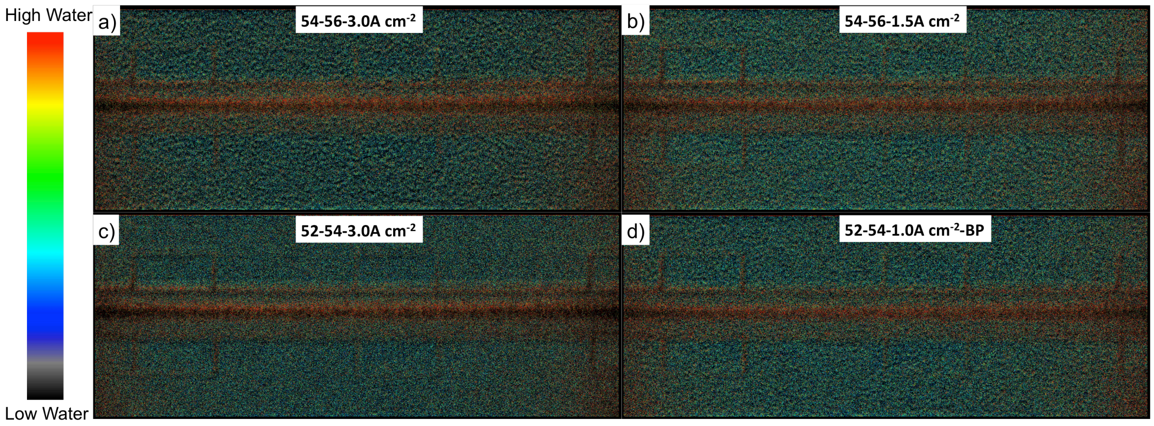

Supplementary Figure 7. The full-length high-resolution images corresponding to Figure 4.

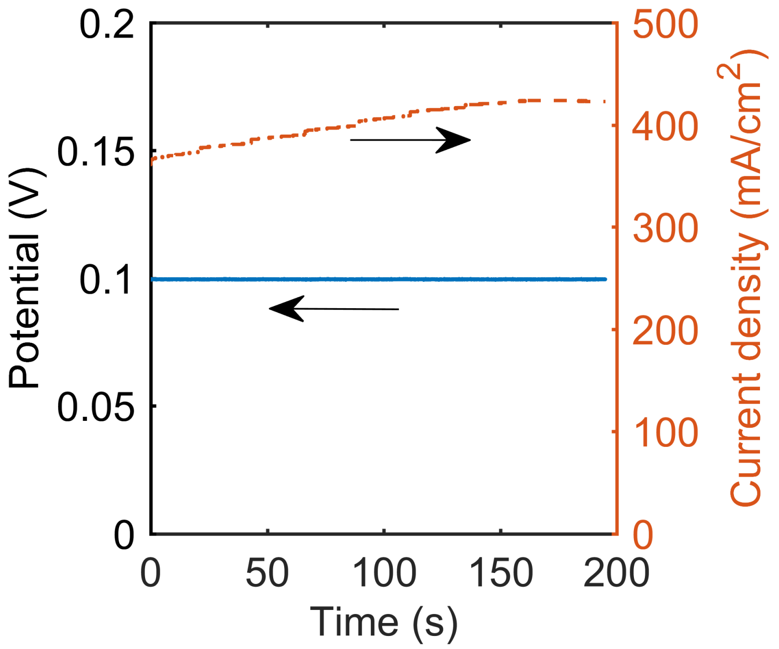

Supplementary Figure 8. The chronoamperometry curve that was recorded during the X-ray CT experiments as shown in Figure 7.

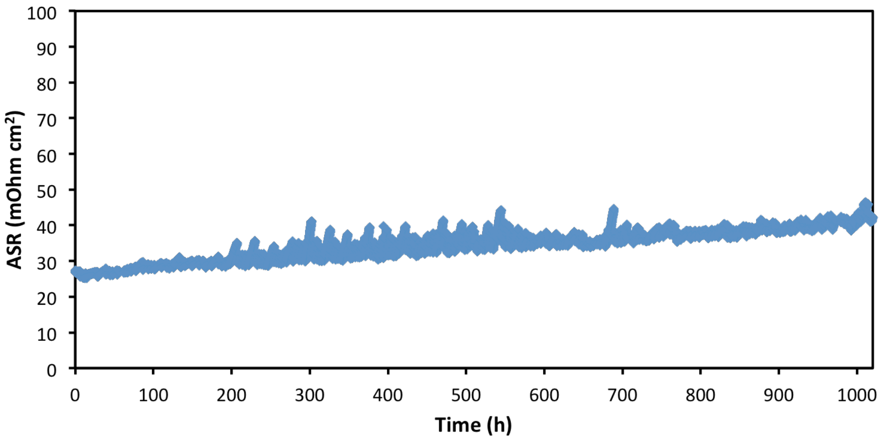

Supplementary Figure 9. The area specific resistance (mOhm cm^2) that was recorded during the 1000h+ stability test (Figure 8).

Supplementary Note 1

Supplementary Figure 1 shows a cross-section tomograph of a typical Alkaline membrane fuel cell (AMFC) under operating conditions. In Supplementary Figure 1a OCV condition at 100 % RH is shown, where ionomer in the anode has already water from water vapor in oxygen. Supplementary Figure 1b shows the cross-section tomograph also at 100 % RH but after several minutes of operation at 200 mA/cm^2. Even at these relatively low current densities we observe substantial water accumulation in anode catalyst layer.

Supplementary Note 2

Previous study reporting polymer electrolyte fuel cells (PEFCs) operando behavior using Swiss Light Source TOMCAT X-ray CT beamline showed significant radiation damage to the PEFC membranes and ionomers at longer scan-times[1]. The study was performed at lower X-ray energy of 13.5 keV, which results in significant amount of X-rays being attenuated by material. Beam damage is reversely proportional to X-ray energy, as with higher energies materials attenuate less X-rays, resulting in lower dosage and damage. At typical photon flux at TOMCAT is 10^{13} photons s^{-1} mm^{-2}. In our study Advanced Light Source (ALS) 8.3.2 X-ray CT beamline was used for measurements. The beamline photon flux is on the order of 10^{11} photons s^{-1} mm^{-2}, two orders of magnitude lower than that at SLS. This is primarily due to lower efficiency optics used at 8.3.2 compared to TOMCAT. Furthermore, 24 keV energy was used for imaging in this study, which is again higher than that used at SLS. We have previously reported imaging of PEFCs from ALS and no significant beam damage was observed[2].

Supplementary Note 3

Supplementary Figure 2 shows water and carbon thickness for X-ray CT operando anodes. Since the carbon fiber content is the same in all three cases the only difference between the three cases is the presence of water. As can be observed 100 % RH and 200 mA/cm^2 operating condition has the highest content of water, whereas at 67 % RH, 0.1 V the cell has less water than at 100 % RH OCV. Furthermore, we observe that on average locations of the anode under the land has less water than the anode locations under the channel. This is due to two reasons, first is that the locations under the land might have lower current densities due to lower gas diffusion an also can be hotter, as they are not convectively cooled.

Supplementary Note 4

Supplementary Figures 3 and 4 show the *ex-situ* X-ray CT images of PtRu/C anode GDE with ETFE ionomer and Pt cathode, respectively. In the grey-scale cross-section tomograph view the catalyst particles show up as bright (due to high attenuation

compared to carbon and ETFE). The three dimensional volume renderings of the GDE show electrode morphology and catalyst distribution. Catalyst agglomeration is observed in some locations at the GDE. The surface roughness present on the surface of the anode will be reduced during assembly of these layers into the MEA, as they will be under compression.

Supplementary Note 5

Supplementary Figure 5 shows polarization curves acquired with micro X-ray CT 1 cm^2 hardware at the beamline, where the cells shows 10 times lower current densities at 67 % RH compared to 100 % RH operating conditions. This is due to the fact that lower dew points are not desirable for low current density cells, as less water is produced in the HOR and more water is needed to enter the cell from the gas stream (shown in Figure 3 in manuscript). For 100 % RH operation the cell maintained constant current density of 200 mA/cm^2 during the scan. However, for 67 % RH, as can be also seen from the polarization curve, the cell could not support 200 mA/cm^2 and we opted to instead apply constant potential of 0.1 V. For the potential the cell generated from 150 to 60 mA/cm^2. Due to limitations of precision of temperature control and operation at ambient pressure, the *operando* fuel cell performance is lower than that achieved at the optimized conditions within 5 cm^2 hardware used in this study. It is important to note that thermal mass of the operando cell is much smaller than conventional fuel cell hardware used for 5 cm^2 active area testing. Active area in operando is not shielded by aluminum plates that are used for compression above and below this area, allowing higher thermal flux out of the cell. Lower thermal mass and unshielded area most likely results in lower temperature in active area than the reading of thermocouples (mounted inside the aluminum plate on the top) provide, which in turn can result in local higher RH and contribute (along with low temperature operation) to the flooding behavior observed by polarization curve (Supplementary Figure 5). Three-dimensional stack of 2000 images is acquired in each X-ray CT scan.

Supplementary Table

Supplementary Table 1. The Anion exchange membranes properties used in this work.

AEM	Water Uptake (%)	Ionic Conductivity At 80 °C (mS cm^{-1})	Ion Exchange Capacity (mmol g^{-1})	Wet Thickness (µm)	Through Plane Swelling (%)
HDPE-based[3]	155±15	214±2	2.44±0.04	30	38±7
ETFE-based[4]	53±6	60±2	2.01±0.02	60±2	28±4

Supplementary References

[1] J. Roth, J. Eller, F.N. Büchi, Effects of Synchrotron Radiation on Fuel Cell Materials, J. Electrochem. Soc. 159 (2012) F449–F455.
[2] S.J. Normile, D.C. Sabarirajan, O. Calzada, V. De Andrade, X. Xiao, P. Mandal, et al., Direct observations of liquid water formation at nano- and micro-scale in platinum group metal-free electrodes by operando X-ray computed tomography, Mater. Today Energy. 9 (2018) 187–197.
[3] L. Wang, X. Peng, W.E. Mustain, J.R. Varcoe, Radiation-grafted anion-exchange membranes: the switch from low- to high-density polyethylene leads to remarkably enhanced fuel cell performance, Energy Environ. Sci. 12 (2019) 1575–1579.
[4] L. Wang, E. Magliocca, E.L. Cunningham, W.E. Mustain, S.D. Poynton, R. Escudero-Cid, et al., An optimised synthesis of high performance radiation-grafted anion-exchange membranes, Green Chem. 19 (2017) 831–843.
[1] J. Roth, J. Eller, F.N. Büchi, Effects of Synchrotron Radiation on Fuel Cell Materials, J. Electrochem. Soc. 159 (2012) F449–F455.
[2] S.J. Normile, D.C. Sabarirajan, O. Calzada, V. De Andrade, X. Xiao, P. Mandal, et al., Direct observations of liquid water formation at nano- and micro-scale in platinum group metal-free electrodes by operando X-ray computed tomography, Mater. Today Energy. 9 (2018) 187–197.
[3] L. Wang, X. Peng, W.E. Mustain, J.R. Varcoe, Radiation-grafted anion-exchange membranes: the switch from low- to high-density polyethylene leads to remarkably enhanced fuel cell performance, Energy Environ. Sci. 12 (2019) 1575–1579.
[4] L. Wang, E. Magliocca, E.L. Cunningham, W.E. Mustain, S.D. Poynton, R. Escudero-Cid, et al., An optimised synthesis of high performance radiation-grafted anion-exchange membranes, Green Chem. 19 (2017) 831–843.
