# Supplementary material for: Using operando techniques to understand and design high performance and stable alkaline membrane fuel cells
Source: Nat Commun. 2020 Jul 16;11:3561. doi: 10.1038/s41467-020-17370-7 (PMC7366663; doi:10.1038/s41467-020-17370-7)
Supplement: Supplementary file 3 — Description of Additional Supplementary Files [file 41467_2020_17370_MOESM3_ESM.docx]

**Description of Additional Supplementary Files**

1. File Name: Supplementary Movie 1

Description: Temporal water distribution across the gas diffusion layers, catalyst layers and membranes in alkaline membrane fuel cells when the anode and cathode gas relative humidity is reduced from 100% to lower relative humidity. 1s in the movie represents about 15 s in real time during measurements. As the relative humidity of the reacting gases is reduced, there is significantly less overall water in the cell, reducing the risk of cell flooding and excessive ionomer swelling. Please play the video to observe the effect.

2. File Name: Supplementary Movie 2

Description: Temporal water distribution across the gas diffusion layers, catalyst layers and membranes in alkaline membrane fuel cells when the anode and cathode backpressure is changed from 140 Kpa to 0 Kpa and then back to 140 Kpa. 1s in the movie represents about 15 s in real time during measurements. The application of back pressure increases the water content in operating cells due to the suppression of convective evaporation, particularly at the anode where water is produced. Please play the video to observe the effect.
