# Supplementary material for: Using operando techniques to understand and design high performance and stable alkaline membrane fuel cells
Source: Nat Commun. 2020 Jul 16;11:3561. doi: 10.1038/s41467-020-17370-7 (PMC7366663; doi:10.1038/s41467-020-17370-7)
Supplement: Supplementary file 2 — Peer Review [file 41467_2020_17370_MOESM2_ESM.pdf]

Editorial Note: This manuscript has been previously reviewed at another journal that is not operating a transparent peer review scheme. This document only contains reviewer comments and rebuttal letters for versions considered at *Nature Communications.*
